# Supplementary material for: Evidence for hidden leprosy in a high leprosy-endemic setting, Eastern Ethiopia: The application of active case-finding and contact screening
Source: PLoS Negl Trop Dis. 2021 Sep 2;15(9):e0009640. doi: 10.1371/journal.pntd.0009640 (PMC8454944; doi:10.1371/journal.pntd.0009640)
Supplement: Attachment — Submitted filename: Response to reviewers.doc [file pntd.0009640.s001.doc]

**Response to reviewers**

**Methods**

**Reviewer 1**

1. This is cross-sectional survey covering a large population. Due to the nature of the disease only few cases confirmed. This figure is very important regarding hidden leprosy. All positive cases were people not seeking medical treatment. This active case finding strategy discover these people with leprosy but not report to health professionals by themselves.

For analysis we used non-parametric statistical tools.

**Reviewer 2**

2. It is an epidemiological study to extent of hidden leprosy in the general population. Although it is observational study, this prevalence is very important compared to prevalence estimated among patients attending health facility through self-report

**Reviewer 3**

3. The methodology used to track the cases/suspects was considering the existing and feasible for application, in the context of resource limited country like Ethiopia. Learning from the successes of other disease prevention/control and improved health service utilization or health-care seeking through the deployment of health extension program in Ethiopia, data generated by HEW are more reliable. Also, HEWs are very close to household and identify their problem closely work with the community. To minimize non response rate data collectors were trained how to identify suspects and to refer difficult to diagnose cases.

- **Technical aspects**
  - Physical examination were described according to the national guideline(line 143-148)
  - Slit smear examination briefly stated (line 154-159), in Ethiopia leprosy diagnosis is mainly made based on clinical examination. However for those suspects difficult to confirm (especially early stage of leprosy) can be supported by bacteriological examination.
  - In the context of this research we prefer to use leprosy case rather than index case. So we define what is leprosy case in the main document and index case is not used(deleted) (line 187-190)
  - Operational definition of others terminologies(PB,MB, disability..) were modified according to the comments(line 191-203)

**Reviewer 4**

4. Map is inserted and further study design and population were recommended

**Results**

**Reviewer 1**

1. Table 1 is modified (separated as commented), total row is indicated, numbers close to one decimal. Figure 1 is removed.
2. Table is used just to show statistical analysis and the variables included, although all independent variables are not statistical significant

**Reviewer 4**

1. We categorize educational level in to two after data collection for statistical analysis purpose. We found the numbers in different educational level like primary (grade 1 to 8^th^), secondary (9^th^ to 12^th^) and college was very small and difficult to analyze them independently. Therefore we merge all educated individual under literate category.
   a. Comparisons with high leprosy endemic country and Ethiopia, in terms of multi-bacillary proportion were not considered in the discussion.
   b. Regarding the identification of co-prevalent cases. We assume HEW are very close to each household and know their health status and expected not to miss them (not supported by research findings). As you stated the reason could be different. The participants were not at home or not willing to be examined by primary health workers. Therefore, it important to ignore the reason in this discussion unless cleary identified.

**Conclusion**

**Reviewer 1**

1. The discussion section is minimized
   a. Strength and limitation is shift up and next to the discussion part
   b. Conclusions and recommendations are explicitly described

**Reviewer 3**

- One important social determinant (poverty& leprosy) (line number 311-315) related the study site is discussed. The limitation of the study were indicated in the document
- The importance (significance) of the current study compared to the current strategy used in Ethiopia ,were addressed in the discussion section (line 383-387).
- Although the sample size we analyzed is small. The finding can give clue for a gap to meet the elimination target (line 279-281). Therefore, this finding should be supported with further studies using larger sample size (345-346).
  - Shortage of MDT may lead to delay related healthy facility. However this issue was discussed with the district authority for better management of leprosy patients. We found that the main reason was about the distribution of the drug, but not because of the shortage of the drugs in the stock.

**Reviewer 4**

- Our survey is based on referring all participants with any skin diseases. These are further examined and confirmed for leprosy by dermatologist. It is an ethical to treat all patients after the diagnosis. Therefore, participants diagnosed as leprosy were initiated MDT and other skin diseases case were linked to regular skin /dermatology clinic. The shortage of MDT were occurred because of unequal distribution of the drugs (administrative problem), which may lead to delay in diagnosis and sever outcome. These issues were discussed and well addressed with the district administrative.
- Participation was fully voluntary. We indicated this part in the ethical issue section(in the document)
- One important social determinant (poverty& leprosy) (line number 311-315) related the study site is discussed. The limitation of the study were indicated in the document
- Recommendation which indicated further study was included.

**Editorial and data presentation**

**Reviewer 1**

1. All kebele changed to village

**Reviewer 4**

2. Table and graph were prepared according to PlOS guidelines

**Summary and general comments**

**Reviewer 1**

The manuscript is shortened and edited for language *by professionals*

***Reviewer 3***

*Images of examined patients were include as supporting document*

***Reviewer 4***

The term elimination was confusing and misleading in leprosy control. Therefore we prefer to remove from the title and it was not the main aim this publication. The main aim of this publication is discovering hidden leprosy through active case finding and household contact tracing.

**Lists of major changes in the manuscript**

1. The manuscript was edited for English language
2. Email address changed
3. Title modified( post-elimination era )
4. Results and discussion only focused to the major finding s to minimize the the size of the manuscript.
5. Map of the study setting was inserted
6. Samples of image of examined patients were included
